# A systematic review and meta-analysis of the association of dietary diversity with undernutrition in school-aged children

**DOI:** 10.1186/s12887-023-04032-y

**Published:** 2023-05-29

**Authors:** Mobina Zeinalabedini, Behzad Zamani, Ensieh Nasli-Esfahani, Leila Azadbakht

**Affiliations:** 1 Department of Community Nutrition, School of Nutritional Sciences and Dietetics, Tehran University of Medical Sciences, PO Box 1416643931, Tehran, Iran; 2 Students’ Scientific Research Center (SSRC), Tehran University of Medical Sciences (TUMS), Tehran, Iran; 3 Diabetes Research Center, Endocrinology and Metabolism Clinical Sciences Institute, Tehran University of Medical Sciences, Tehran, Iran; 4 Department of Community Nutrition, School of Nutrition and Food Science, Isfahan University of Medical Science, Isfahan, Iran

**Keywords:** Dietary diversity score (DDS), Undernutrition, School-aged children, Stunting, Thinness

## Abstract

**Background:**

Malnutrition in childhood has lasting consequences; its effects not only last a lifetime but are also passed down from generation to generation such as short stature, school-aged children are the most vulnerable section of the population and require special attention, including nutrition.

**Method:**

We searched Medline through PubMed, Scopus, and Web of Science to identify all observational studies published before Jun 2022. Observational studies with a pediatric population aged 5–18 years that evaluated risk estimate with 95% confidence intervals the relationship between dietary diversity and undernutrition (wasting, stunting, and thinness) were included. The Preferred Reporting Items for Systematic Reviews and Meta-analyses (PRISMA) were followed.

**Results:**

This is a first systematic review and meta-analysis with a total of 20 studies were eligible (n = 18 388). Fourteen data evaluated stunting resulting in a pooled effect size estimated odds ratio of 1.43 (95% CI: 1.08–1.89; p = 0.013). Ten data evaluated Thinness resulting in a pooled effect size estimated odds ratio of 1.10 (95% CI: 0.81–1.49; P = 0.542). Two studies were revealed wasting with a odds ratio of 2.18 (95% CI: 1.41–3.36; p-value < 0.001).

**Conclusion:**

According to the conclusions of this meta-analysis of cross-sectional studies, inadequate dietary diversity increases the risk of undernutrition in growth linear but not in thinness in school-aged children. The findings of this analysis suggest that initiatives that support improvements to the diversity of children’s diets to reduce the risk of undernutrition may be warranted in LMICs.

**Supplementary Information:**

The online version contains supplementary material available at 10.1186/s12887-023-04032-y.

## Introduction

Malnutrition is a term that refers to energy or nutritional deficiency, as well as excess or imbalanced nutrition intake. There are three distinct forms of malnutrition: (1) malnutrition due to micronutrient deficiency, (2) malnutrition due to micronutrient deficiency and obesity, (3) undernutrition classified as wasting (low weight-to-height ratio), stunting (low height-to-age ratio), and underweight (low weight-for-age) [1].

Malnutrition in childhood has lasting consequences; its effects not only last a lifetime but are also passed down from generation to generation, such as; high absenteeism, early dropout, unsatisfactory classroom performance, delayed cognitive development, short stature, reduced work capacity, and poor reproductive performance and health problem that are among the most common causes of low school enrolment [2–4]. School age is a time of significant physical, mental, and emotional development for children in this age group [5]. Their social engagement also expands beyond their immediate family, posing an additional risk due to less nutritional care and support. As with children under five, school-aged children are the most vulnerable section of the population and require special attention, including nutrition [1, 6]. Adolescent females are more susceptible than boys due to their specific physiological traits, such as menarche, and deep-rooted gender norms leading to intra-household disparities in food consumption [7].

Thinness has been embraced as a more accurate predictor of recent nutritional deprivation in older children, such as insufficient energy, protein, or micronutrients, than underweight [8]. According to the date data revealed in 2022, 21% of school-aged children were stunted, 12.5% were thin, and 24% were wasted [9]. Despite economic growth in emerging countries, malnutrition is widespread [10]. Children in low- and middle-income nations have a higher risk of malnutrition due to poverty and a lack of food [11]. Children’s physiology and the family and community’s effect on their behavior may all play a role in the child’s optimal growth and nutritional status [12].

A dietary variety score, a simple count of food types consumed by an individual over the preceding 24 h, is regarded as a proxy for an individual’s nutrient-adequacy diet [13]. Insufficient nutritional intake among school-aged children may have short-term and long-term detrimental effects on health, including postponed physical development and impaired cognitive development in adolescence [14], and an elevated risk of cardiometabolic disorders in adulthood [15].

Dietary variety is a crucial sign of a high-quality diet and is especially important for school-aged children whose bodies have high nutrient needs [16]. Numerous studies conducted in African countries have demonstrated that a lack of dietary diversity relates to malnutrition, whereas a diverse diet can enhance overall nutritional status [17–19]. However, some studies have not observed any association between dietary diversity and undernutrition [20–22].

Therefore, this systematic review and meta-analysis of published cross-sectional studies synthesized data about the connection between diet diversity and the risk of wasting, stunting, and thinness in school-aged children because to the best of our knowledge no study has explained this debate.

## Method

This study complied with the Preferred Reporting Items for Systematic Reviews and Meta-analyses (PRISMA) guidelines [23]. Data were double-screened for full-text publications, and two reviewers worked separately from selection through data extraction (MZ and BZ). A third reviewer resolved any issues that arose (LA).

### Search strategy

We carefully searched MEDLINE (through PubMed), Scopus, and Web of Science without regard for language restrictions until Jun 8th, 2022. Dietary diversity, DDS, Dietary diversity score, wasting, underweight, and stunting (Table S1) were used in the search approach. All relevant papers were identified by searching published reference lists, and the corresponding author was contacted via email to request access to the full study text. All publications were kept in an EndNote library (version 20 for Windows) to facilitate the referral process.

### Inclusion and exclusion criteria

To set inclusion criteria, two independent reviewers examined all fields of research discovered by the search strategy, which were as follows: (1) children over the age of five, (2) observational design studies (cross-sectional, cohort, or case-control studies), and (3) reported hazard ratios (HR), relative risks (RR), or odds ratios (OR) with 95% confidence intervals (CI) to determine the relationship between dietary diversity and the risk of wasting, stunting, and thinness. These were the exclusion criteria: (1) children under the age of 5, (2) maternal or paternal dietary variety, (3) animal studies, (4) not in English, (5) review articles, opinions, editorials, or letters, as well as unpublished studies or abstracts, (6) Intervention studies, (7) populations with acute diseases such as cancer, cystic fibrosis, and others, and (8) statistical analyses revealed correlation coefficients rather than estimated risk.

### Study selection

The screening process for studies was conducted independently by two reviewers, as previously reported. Two reviewers first did a title-abs screening, and then the remaining titles likely to be included were reviewed in full-text so that no relevant work was overlooked. Two reviewers independently did full text screening on every potentially relevant paper discovered.

### Data extraction

In addition to the impact size estimations, the following factors were gathered: (1) Child age range (years), (2) child gender, (3) dietary measurement, (4) the number of participants, (5) research publication year, (6) design of the study (cross-sectional or longitudinal), (7) quality of studies, (8) country of origin, and (9) any modifications to their study.

If two reports utilized the same data set, we chose the one with the greatest sample size and the statistical analysis. We utilized the adjusted measure if both unadjusted and adjusted effect sizes were available.

### Quality assessment

Two reviewers assessed the relevant cross-sectional studies using the updated Newcastle–Ottawa quality assessment scale (Table S2).

Quality of studies was assessed as follows: poor (3), reasonable (4–6), and strong (7). The revised Newcastle–Ottawa quality rating scale for cross-sectional research awarded a maximum score of 10 to cross-sectional studies. This is a representative tool used for observational studies to evaluate studies in three sections: study selection, comparability, and outcome. The overall score was measured by summarizing each score.

### Grading the evidence

We evaluated the overall degree of confidence in the evidence for each relationship using the revised Grading of Recommendations, Assessment, Development, and Evaluations (GRADE) method. GRADE evaluations were carried out separately by the authors (MZ and BZ).

### Data analysis

Risk estimates for undernutrition were calculated in two ways: using the dietary diversity score (DDS; greatest score vs. lowest score) or using the minimal diet diversity (MDD). The relevant SEs of ORs 24 were then determined using the method SE log (RR) = [SElog (OR) log (RR)]/log (OR). The natural logarithms of the ORs, as well as their SEs and 95% CIs, were calculated.

To examine homogeneity, the I^2^ Index, Cochran’s Q statistic, and the associated P-value for heterogeneity were utilized. To detect publication bias, visual inspection of funnel plots and Egger’s tests for funnel plot asymmetry were performed. We were able to investigate consistency in the pooled data by removing one trial at a time and re-estimating the pooled OR. All statistical analyses were performed using STATA 11.0 software (STATA Corp.). Statistical significance was defined as P values less than 0.05 [24].

## Results

**Fig. 1:**
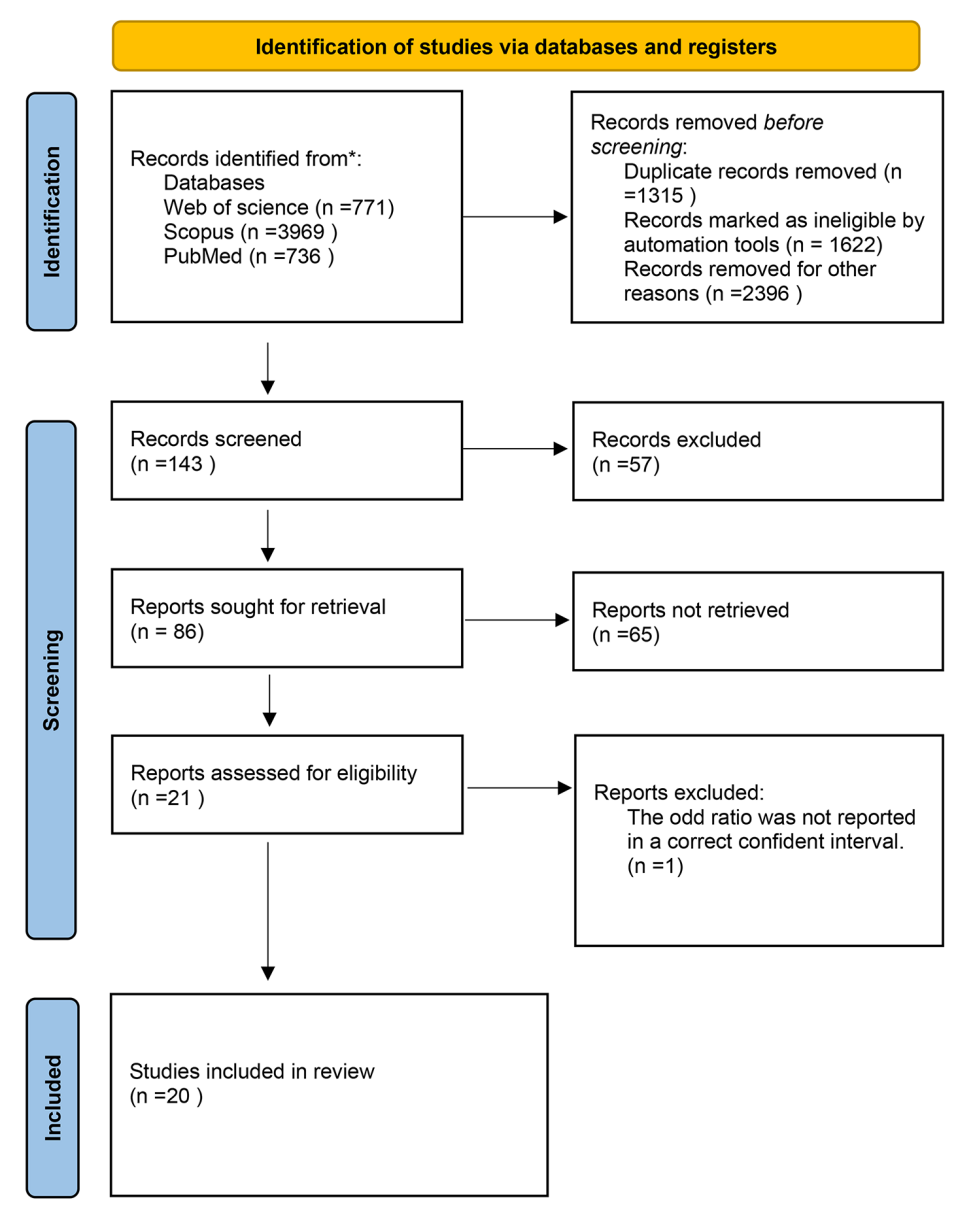
PRISMA diagram of selection process

The flow diagram depicts the search strategy and results (Fig. 1). During the first search, 5478 studies were discovered. A full-text review of 143 articles was conducted. The final analysis comprised 20 studies ranging in age from 5 to 18 years.

### Systematic review

The review examined the following data released between 2011 and 2022: 2011 (n = 1), 2013 (n = 1), 2015 (n = 1), 2017 (n = 1), 2018 (n = 2), 2019 (n = 4), 2020 (n = 5), 2021 (n = 3), 2022 (n = 1). The features of the included studies are shown in Table 1. Among the studies included, six were done on girls, while the remaining were conducted on both sexes. Except for seven studies performed in other countries (Madagascar (n = 1) [25], India (n = 2) [26, 27], Sri Lanka (n = 2) [28, 29], Cambodia (n = 1), and Nigeria (n = 1)), the rest were conducted in Ethiopia. Except for two studies that used 7-day recall [20] and 30 days-FFQ [30], and one study did not reveal the dietary assessment index [31], the reminder utilized 24-hour recall for dietary assessment. With only three studies with HIV-positive children [21, 31, 32], all the studies investigated healthy children’s populations. Stunting, wasting, and thinness was identified using a height-for-age z-score (HAZ) of -2 SD, a weight-for-length z-score (WLZ) of -2 SD, and a weight-for-age z-score (WAZ) of -2 SD SDs below the WHO population median, respectively.

**Table 1:** Summary Characteristics of Included Studies

Author, Year	Country	Age	Sex	Health status	Sample size	Diet index assessment	Comparison	No. of variables in adjustments	Outcome	Quality score
Adeomi et al. 2022 [30]	Nigeria	6–19 years old	G/B	Healthy	1200	30 days-FFQ	DDS < 4 food groups VS. DDS ≥ 4 food groups	5	Thinness	10
Mulu Birru et al. 2021 [41]	Ethiopia	14–15 years old	G/B	Healthy	364	24-h recall	DDS ≥ 5 food groups vs. DDS < 5 food groups	26	Stunting	10
Mersha et al. 2021 [17]	Ethiopia	10–19 years old	G	Healthy	706	24-h recall	DDS ≥ 5 food groups vs. DDS < 5 food groups	12	Stunting/ Wasting	10
Patil et al. 2021 [27]	India	16–18 years old	G	Healthy	586	24-h recall	DDS < 3 food groups vs. DDS ≥ 3 food groups	NOT Adjusted	Stunting/ Thinness	7
Yasuoka et al. 2020 [21]	Ethiopia	6–15 years old	G/B	HIV	298	24-h recall	DDS 5–7 groups vs. DDS 0–4 groups	10	Stunting/ Wasting	8
Shiferaw et al. 2020 [32]	Ethiopia	10–19 years old	G/B	HIV	260	24-h recall	DDS < 6 food groups vs. DDS ≥ 6 food groups	NOT Adjusted	Stunting/ Thinness	7
Kahssay et al. 2020 [18]	Ethiopia	10–19 years old	G	Healthy	340	24-h recall	DDS < 4 food groups vs. DDS ≥ 4 food groups	10	Stunting/ Thinness	10
Gezahegn et al. 2020 [31]	Ethiopia	5–15 years old	G/B	HIV	405	Not reported	DDS < 4 food groups vs. DDS ≥ 4 food groups	5	Stunting	10
Tariku et al. 2019 [55]	Ethiopia	10–19 years old	G	Healthy	1550	24-h recall	DDS < 4 food groups vs. DDS ≥ 4 food groups	NOT Reported	Stunting	7
Jikamo et al. 2019 [20]	Ethiopia	13–17 years old	G/B	Healthy	2084	7days-recall	DDS$\le 4$food grups DDS > 9–12 food groups	S: 6 T:7	Stunting	10
Getaneh et al. [19] 2019	Ethiopia	6–14 years old	G/B	Healthy	523	24-h recall	DDS ≥ 7 food groups vs. DDS < 4 food groups	**4**	Stunting	10
Engidaw et al. 2019 [56]	Ethiopia	10–19	G	Healthy	423	24-h recall	DDS$\le 3$food grups vs. DDS > 6food groups	**4**	Stunting	8
Belay et al. 2019 [48]	Ethiopia	5–14 years old	G/B	Healthy	848	24-h recall	DDS < 4 food groups vs. DDS ≥ 4 food groups	**4**	Thinness	8
Aiga et al. 2019 [25]	Madagascar	5–14 years old	G/B	Healthy	205	24-h recall	DDS$\le 4$food grups vs. DDS > 9–12 food groups	**16**	Thinness	10
Tariku et al. 2018 [57]	Ethiopia	6–14 years old	G/B	Healthy	389	24-h recall	DDS < 4 food groups vs. DDS ≥ 4 food groups	**4**	Stunting/ Thinness	10
Radhika et al. 2018 [26]	India	10–19 years old	G	Healthy	3930	24-h recall	DDS > 5 food groups vs. DDS ≤ 5 food groups	NOT Reported	Thinness	8
Getachew et al. 2017 [58]	Ethiopia	7–14 years old	G/B	Healthy	511	24-h recall	DDS < 4 food groups vs. DDS ≥ 4 food groups	**4**	Stunting	10
Wassie et al. 2015 [28]	Sri Lanka	10–19 years old	G	Healthy	1281	24-h recall	DDS < 3 food groups vs. DDS > 6 food groups	**4**	Thinness	10
Darapheak et al. 2013 [59]	Cambodia	6–14 years old	G/B	Healthy	523	24-h recall	DDS ≥ 7 food groups vs. DDS < 4 food groups	**8**	Stunting	9
Niranjala et al. 2011 [29]	Sri Lanka	13–16 years old	G/B	Healthy	205	24-h recall	DDS > 5 food groups vs. DDS ≤ 5 food groups	**10**	Thinness	9

MDD: Minimum Dietary Diversity, DDS: Dietary Diversity Score, S: Stunting, W: Wasting, B: boy, G: girl

Fourteen effect sizes reported stunting, ten effect sizes reported thinness, and only two studies revealed wasting.

### Quality assessment

According to our findings, all the included studies received a high-quality score (Table S2). As previously described, we used the NOS scale to assess the quality of studies and sum up the total score above 7.

### Meta-analyses

#### Dietary diversity and stunting

**Fig. 2:**
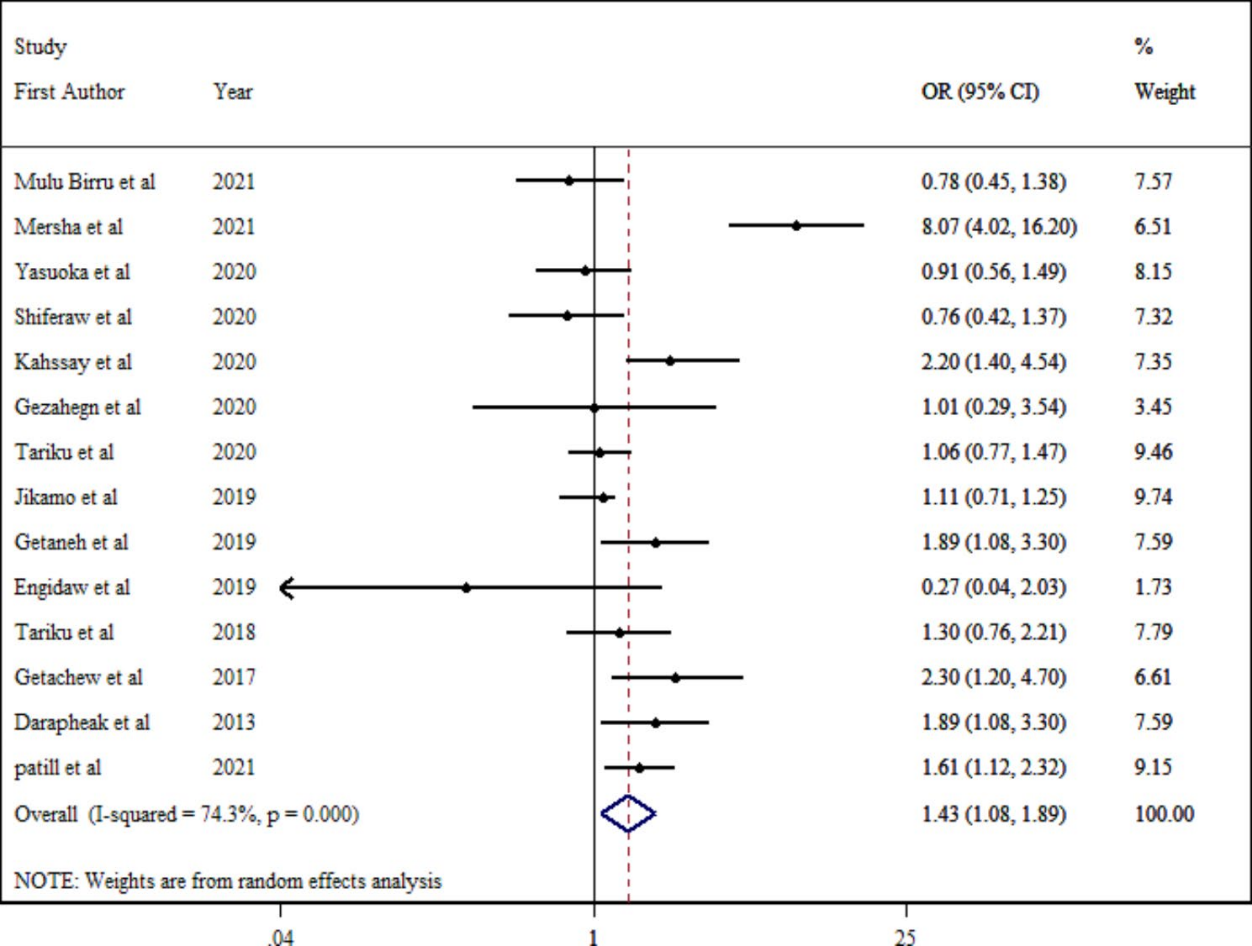
Estimated odds of stunting among school-aged children

Fourteen effect sizes were included in the final analysis with 8539 individuals. Participants with inadequate dietary diversity had a 43% higher (pooled OR: 1.43; 95% CI: 1.08–1.89; p = 0.013) estimated odds of stunting compared to those with adequate diet diversity (Fig. 2). Due to considerable between-study heterogeneity (${I}^{2}$ = 74.3%; P_Q−test_ <0.001), subgroup analysis was conducted based on different parameters (Table 2). According to the stratified analysis by location, the pooled estimates for the association between dietary diversity and stunting remained significant in studies conducted in the Asian continent (OR:1.69; 95% CI: 1.24–2.29; p-value < 0.001), studies with a more than 500 population (OR:1.86; 95% CI: 1.25–2.78; p-value = 0.002), and studies were conducted on healthy individuals (OR:1.61; 95% CI: 1.17–2.21; p-value = 0.004). In addition, subgroup analysis indicated that heterogeneity was removed in studies with less equal than 500 individuals (${I}^{2}$ = 45.9%; P_Q−test_ =0.086), and studies were conducted in both groups of sex (${I}^{2}$ = 46.0%; P_Q−test_ =0.063).

**Table 2:** Stratified analysis of the association between dietary diversity and undernutrition

	Number of studies		Odds Ratio (95% CI)		*P* value		*P*-heterogeneity		*I*^*2*^ (%)	
	Stunting	Thinness	Stunting	Thinness	Stunting	Thinness	Stunting	Thinness	Stunting	Thinness
**Overall**	14	10	1.43 (1.08–1.89)	1.10 (0.81–1.49)	< 0.001	0.542	< 0.001	< 0.001	74.3%	79.9%
**Sample size**										
≤ 500	7	5	1.04 (0.73–1.47)	1.05 (0.68–1.64	0.834	0.821	0.086	0.002	45.9%	76.5%
> 500	7	5	1.86 (1.25–2.78)	1.13 (0.70–1.84)	0.002	0.611	< 0.001	0.001	82.4%	83.4%
**Continent**										
Africa	12	7	1.37 (0.98–1.92)	1.20 (0.81–1.77)	0.061	0.365	< 0.001	< 0.001	76.8%	81.8%
Asia	2	3	1.69 (1.24–2.29)	0.89 (0.81–1.49)	< 0.001	0.718	0.637	0.003	0.0%	82.8%
**Sex**										
Girl	9	4	1.87 (0.94–3.71)	1.16 (0.57–2.36)	0.117	0.684	< 0.001	< 0.001	87.2%	87.5%
Both	5	6	1.22 (0.95–1.56)	1.06 (0.76–1.48)	0.075	0.714	0.063	0.002	46.0%	73.0%
**Health status**										
Healthy	11	9	1.61 (1.17–2.21)	1.09 (0.79–1.51)	0.004	0.604	< 0.001	< 0.001	77.1%	82.0%
HIV	3	1	0.86 (0.60–1.23)	1.18 (0.61–2.29)	0.407	0.624	0.868	-	0.0%	-

We visually inspected the funnel plot for asymmetry for stunting and dietary diversity. The funnel plot (Figure S1) appeared significant for publication bias (Egger test intercept; p = 0.001).

#### Dietary diversity and thinness

**Fig. 3:**
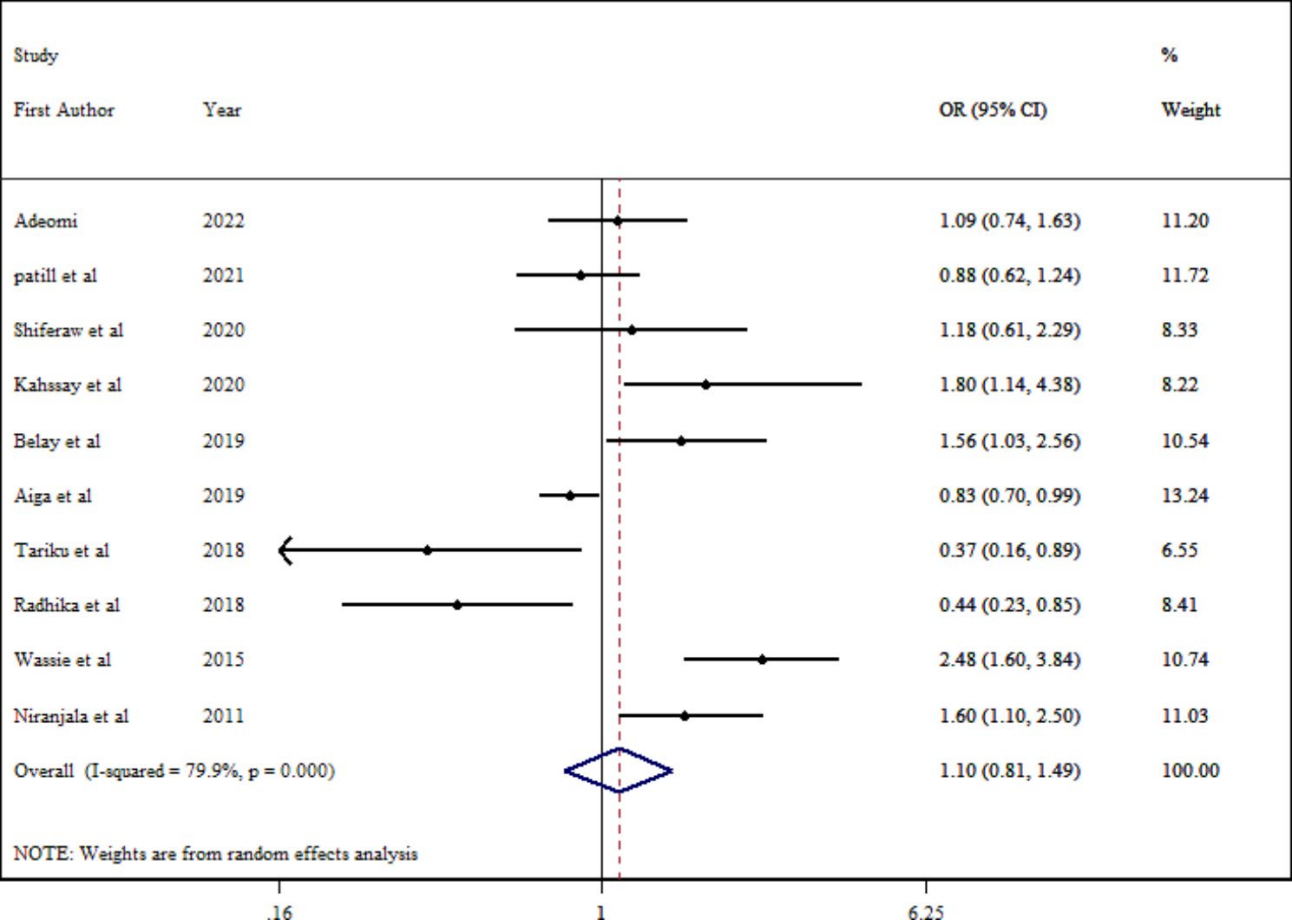
Estimated odds of thinness among school-aged children

Ten effect sizes were included in the final analysis with 9434 individuals that failed to establish a statistically significant association (pooled OR = 1.10; 95% CI: 0.81–1.49; P = 0.542) (Fig. 3). There was considerable heterogeneity among studies (${I}^{2}$ = 79.9%; P_Q−test_ <0.001). Due to considerable between-study heterogeneity, Subgroup analysis was conducted based on different parameters (Table 2). According to the stratified analysis, the pooled estimates for the association between dietary diversity and stunting thinness remained insignificant in all groups. Moreover, sample size, continent, health status, and sex subgroups were identified as a source of heterogeneity. We were able to estimate the funnel plot’s asymmetry based on a visual examination of stunting thinness and dietary diversity. The funnel plot (Figure S2) was not significant for publication bias (Egger test intercept; p = 0.121).

### Dietary diversity and wasting

**Fig. 4:**
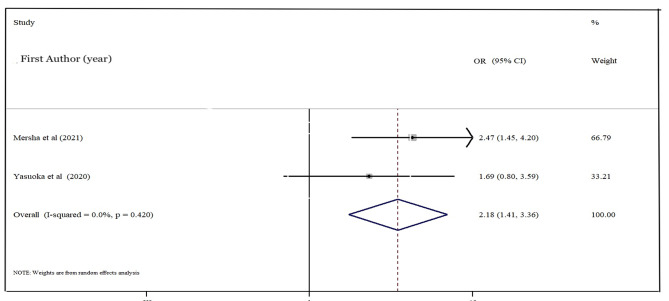
Estimated odds of wasting among school-aged children

Two studies were included in the final analysis with 1004 individuals. According to the result, the participant with the inadequate dietary diversity had two times higher (pooled OR: 2.18; 95% CI: 1.41–3.36; p-value < 0.001) (Fig. 4).

### Sensitivity analysis

Sensitivity analyses were performed for evaluated effect sizes (i.e., ORs for stunting and thinness) by removing data from the meta-analytic model. There was no sensitivity among all studies (Figure S3, S4).

### Certainty of evidence

GRADE was performed for evaluated the certainty of studies. The result of GRADE showed that the certainty of both outcome stunting and thinness is very low while wasting is moderate (Table S3).

## Discussion

Concerning survey data, our meta-analysis and systematic evaluation of cross-sectional research comprised 18,388 participants from 20 studies. According to the findings of this study, school-aged children with a limited diet are more likely to have a higher estimated pooled risk of stunted growth and wasting. However, there is no association between a limited diet and thinness.

Dietary Diversity (DD) is the variety of foods or food categories ingested throughout a certain time period [33]. Diverse foods are the greatest way to guarantee dietary sufficiency and are an excellent source of macro and micronutrients [33]. Dietary variables are linked to an increased risk of chronic illnesses and malnutrition, and both international and national recommendations call for increasing dietary variety [33]. Dietary diversity is vital for satisfying energy and other basic nutritional needs, especially for individuals at risk of nutrient deficiencies, such as children from low-income families. Inadequate dietary variety and higher nutritional needs have been linked to an increased risk of poor development trajectory in children, which becomes more obvious as they grow [34–36].

School-aged is a period of great change [37]. Depending on socioeconomic factors, the transformation could take several years [37–39]. Even within a particular culture, adolescents are not a uniform population; their growth, maturity, and lifestyles vary widely [37–39].

A lack of variety in one’s diet has been linked to an increased risk of short height in adolescents. This might be due to a cereal-based monotonous diet with inadequate quality, amount, and frequency of feeding that does not meet micronutrient needs for child development, such as iron, vitamin B12, folate, and other necessities [40]. In addition, it may be due to low nutrient intake during infancy and childhood that substantially influences the linear growth of the prescribed height as the relevant age [22]. Chronic and cumulative food shortages, lack of essential medication and fuel, infrastructure damage from conflicts, and poor economic conditions may pose significant problems to the nutritional health of teenage in later life [22].

Due to our analysis, dietary diversity has not been linked to thinness which is different from another study that discovered low dietary diversity, three times more likely to become thin [41]. This might be because of insufficient data on the thinness we have had. Studies have shown that nutritional deficiencies due to loss of menstruation, irregular eating habits, and food preferences are common causes of underweight girls in transition [42, 43]. This might be due to changes in eating habits due to menstrual signs and symptoms, peer influence on food preferences, and the presence of monotonous family nutrition due to common rations may contribute to reduced weight gain, and directly affect BMI in adolescent girls [22].

Due to rapid development and increasing nutritional needs throughout adolescence, a low frequency of meals will cause them to become underweight or thin [41]. School-aged children’s thinness is strongly linked to food insecurity at home [44, 45]. Household food insecurity may be linked to a lack of food variety, translating to an insufficient supply of all necessary nutrients [46]. This is consistent with cross-sectional research on 2016 children in Bangladesh [41] and 656 homes with young women in Kenya [42]. Dietary diversity can be used to diagnose food insecurity, according to the FANTA experiment [47]. However, our study confirmed the link between food variety and stunting; a survey in Jimma is in a different line from our research [20].

However, our stratified analysis failed to show a significant association between sex and stunting and thinness, other studies indicated that stunting is significantly higher in female adolescents [20, 48, 49]. This might be due to variation in maturation time in boys and girls, for which girls reached maturation earlier than boys [49]. Adolescence is a period of significant physiological, sexual, neurological, and behavioral changes, and it provides the groundwork for assuming adult tasks and responsibilities, including the move to employment and financial independence, as well as the establishment of lifelong relationships [50].

As this duration is a phase of fast growth, proper nutrition is essential to reach full growth potential, and failure to acquire appropriate food may result in delayed and stunted linear growth and poor organ remodeling [51]. The duration and length of body composition changes are intimately related to sexual development; hence, dietary requirements rely more on sexual maturity than on chronological age [52].

Our stratified analysis showed a significant relationship between stunting and studied conducted in Asia. This might be because more low- and middle-income countries (LMICs) are from the Asian continent, and various research from LMICs have shown a lack of nutritional variety and inadequacy [53, 54]. Since that short stature and HAZ are indicators of a child’s long-term nutritional status, children living in these countries with persistent micronutrient deficiencies were more likely to be stunted.

The current studies have many strengths. To the best of our knowledge, this is the first systematic review and meta-analysis to evaluate the risk estimations for the relationship between dietary variety and undernutrition in school-aged children, and both groups of sex were included. The meta-analysis had a reasonable sample size, indicating that it improved the power of the meta-analysis to detect meaningful conclusions and precise estimates. Finally, all studies included in this review were deemed high-quality based on the assessment tool.

However, limitations should be considered when evaluating the findings of this meta-analysis. We used dietary diversity as a proxy for the overall nutritional quality of the child’s diet. Since this study did not account for the number of foods consumed, it is impossible to determine with certainty if the foods consumed corresponded to the recommended dietary intake. Considerable heterogeneity was found in the primary analysis. However, a subgroup-stratified analysis was performed to evaluate potential causes for heterogeneity. Most studies used a 24-hour dietary recall assessment, which is subject to erroneous categorization and reporting bias and may not capture all relevant dietary data. Finally, all studies were cross-sectional, meaning causality cannot be established, and there remains a limited understanding of the longitudinal relationship between dietary diversity and undernutrition.

## Conclusion

According to the conclusions of this meta-analysis of cross-sectional studies, inadequate dietary diversity increases the risk of undernutrition in growth linear and wasting but not in thinness in school-aged children. The findings of this analysis suggest that initiatives that support improvements to the diversity of children’s diets to reduce the risk of undernutrition may be warranted in LMICs. However, future research, specifically longitudinal cohorts, and clinical trials, with age-classification are needed to confirm these findings and determine the mechanisms responsible for the association between dietary diversity and undernutrition.

## Electronic supplementary material

Below is the link to the electronic supplementary material.

Supplementary Material 1

## Data Availability

The [supplementary Material] data used to support the findings of this study are included within the article.

## References

[1] Available from: https://www.who.int/news-room/fact-sheets/detail/malnutrition.

[2] Dewey KG, Begum K (2011). Long-term consequences of stunting in early life. Matern Child Nutr.

[3] Global, *regional, and national comparative risk assessment of 79 behavioural, environmental and occupational, and metabolic risks or clusters of risks, 1990–2015: a systematic analysis for the Global Burden of Disease Study 2015* Lancet, 2016. 388(10053): p. 1659–1724. 10.1016/S0140-6736(16)31679-8 PMC5388856 27733284

[4] Black RE (2013). Maternal and child undernutrition and overweight in low-income and middle-income countries. Lancet.

[5] Srivastava A (2012). Nutritional status of school-age children - a scenario of urban slums in India. Arch Public Health.

[6] *The Federal Democratic Republic of Ethiopia, National School Health and Nutrition Strategy. Ministry of Education. 2012*; Available from: https://planipolis.iiep.unesco.org/sites/default/files/ressources/ethiopia_national_school_health_nutrition_strategy.pdf.

[7] Chaparro, C., Oot, L. and Sethuraman, K. *Nutrition Profile Nutrition Profile*. 2014; Available from: https://www.fantaproject.org/sites/default/files/download/Laos-Nutrition-Profile-Apr2014.pdf.

[8] *Physical status: the use and interpretation of anthropometry. Report of a WHO Expert Committee*. World Health Organ Tech Rep Ser, 1995. 854: p. 1–452. 8594834

[9] Khan, D.S.A., et al., *Nutritional Status and Dietary Intake of School-Age Children and early adolescents: systematic review in a developing Country and Lessons for the global perspective*. Frontiers in Nutrition, 2022. 8. 10.3389/fnut.2021.739447 PMC8848764 35187014

[10] Müller O, Krawinkel M (2005). Malnutrition and health in developing countries. Cmaj.

[11] *World Health Organization (WHO) ‘Obesity and overweight*. 2011; Available from: https://www.who.int/news-room/fact-sheets/detail/obesity-and-overweight.

[12] Liu, J. and A. Raine, *Nutritional status and social behavior in preschool children: the mediating effects of neurocognitive functioning*. Matern Child Nutr, 2017. 13(2). 10.1111/mcn.12321 PMC5675074 27133006

[13] Yvette Fautsch Macías, P.G., *Guidelines for assessing nutrition-related knowledge, attitudes and practices*. 2014.

[14] Patton GC (2016). Our future: a Lancet commission on adolescent health and wellbeing. Lancet.

[15] Victora CG (2008). Maternal and child undernutrition: consequences for adult health and human capital. Lancet.

[16] Verger EO (2021). Dietary diversity indicators and their Associations with Dietary Adequacy and Health Outcomes: a systematic scoping review. Adv Nutr.

[17] Mersha, J., A. Tariku, and K.A. Gonete, *Undernutrition and Associated factors among School adolescent girls attending schools in Mirab-Armachiho District, Northwest Ethiopia*. Ecology of Food and Nutrition, 2021. 10.1080/03670244.2021.1872022 33426928

[18] Kahssay, M., L. Mohamed, and A. Gebre, *Nutritional Status of School Going Adolescent Girls in Awash Town, Afar Region, Ethiopia* Journal of Environmental and Public Health, 2020. 2020. 10.1155/2020/7367139 PMC7054789 32148529

[19] Getaneh, Z., et al., *Prevalence and determinants of stunting and wasting among public primary school children in Gondar town, northwest, Ethiopia*. BMC Pediatrics, 2019. 19(1). 10.1186/s12887-019-1572-x PMC6591879 31238889

[20] Jikamo, B. and M. Samuel, *Does dietary diversity predict the nutritional status of adolescents in Jimma Zone, Southwest Ethiopia?* BMC Research Notes, 2019. 12(1). 10.1186/s13104-019-4437-3 PMC6628467 31307544

[21] Yasuoka, J., et al., *Nutritional status and dietary diversity of school-age children living with HIV: a cross-sectional study in Phnom Penh, Cambodia*. BMC Public Health, 2020. 20(1). 10.1186/s12889-020-09238-8 PMC7388459 32727433

[22] Engidaw, M.T. and A.D. Gebremariam, *Prevalence and associated factors of stunting and thinness among adolescent somalian refugee girls living in eastern somali refugee camps, somali regional state, Southeast Ethiopia*. Conflict and Health, 2019. 13(1). 10.1186/s13031-019-0203-3 PMC6525407 31131019

[23] Page MJ (2021). The PRISMA 2020 statement: an updated guideline for reporting systematic reviews. BMJ.

[24] Boston R, Sumner A (2003). STATA: a statistical analysis system for examining Biomedical Data. Advances in experimental medicine and biology.

[25] Aiga, H., et al., *Risk factors for malnutrition among school-aged children: a cross-sectional study in rural Madagascar*. BMC Public Health, 2019. 19(1). 10.1186/s12889-019-7013-9 PMC6580631 31208397

[26] Radhika, M.S., et al., *Dietary and nondietary determinants of nutritional status among adolescent girls and adult women in India*, in *Annals of the New York Academy of Sciences*. 2018. p. 5–17.

[27] Patil, S., et al., *Stunting is A Reflection of Poor Dietary Diversity Among Adolescent Girls in Rural KONKAN Region (DERVAN-6)* 2021.

[28] Wassie, M.M., et al., *Predictors of nutritional status of Ethiopian adolescent girls: a community based cross sectional study*. BMC Nutrition, 2015. 1(1).

[29] Niranjala AMS, Gunawardena NS, Infant (2011). Nutritional status of adolescent females in estates in Haliela, Sri Lanka. Child, & Adolescent Nutrition.

[30] Adeomi, A.A., A. Fatusi, and K. Klipstein-Grobusch, *Food Security, Dietary Diversity, dietary patterns and the double burden of malnutrition among school-aged children and adolescents in two nigerian States*. Nutrients, 2022. 14(4). 10.3390/nu14040789 PMC8875779 35215439

[31] Gezahegn D (2020). Predictors of stunting among pediatric children living with HIV/AIDS, Eastern Ethiopia. International Journal of Public Health Science.

[32] Shiferaw H, Gebremedhin S (2020). Undernutrition among HIV-Positive adolescents on antiretroviral therapy in Southern Ethiopia. Adolesc Health Med Ther.

[33] Kennedy, G., T. Ballard, and M.C. Dop, *Guidelines for measuring household and individual dietary diversity*. 2011: Food and Agriculture Organization of the United Nations.

[34] Wondimagegne Z (2020). Child feeding practice and primary Health Care as Major Correlates of Stunting and Underweight among 6- to 23-Month-Old Infants and Young Children in Food-Insecure Households in Ethiopia. Current developments in nutrition.

[35] Islam AHMS (2018). Farm diversification and food and nutrition security in Bangladesh: empirical evidence from nationally representative household panel data. Food Security.

[36] Msc B (2001). Significance of wild vegetables in micronutrient intakes of women in Vietnam: an analysis of food variety. Asia Pacific Journal of Clinical Nutrition.

[37] Jaworska N, MacQueen G (2015). Adolescence as a unique developmental period. J Psychiatry Neurosci.

[38] Valentine G (2003). Boundary crossings: transitions from Childhood to Adulthood. Children’s Geographies.

[39] Akseer, N., et al., *Global and regional trends in the nutritional status of young people: a critical and neglected age group*, in *Annals of the New York Academy of Sciences*. 2017. p. 3–20. 10.1111/nyas.13336 28436100

[40] Eicher-Miller HA (2009). Food insecurity is associated with iron deficiency anemia in US adolescents. Am J Clin Nutr.

[41] Mulu Birru, G., et al., *Malnutrition in School-Going Adolescents in Dessie Town, South Wollo, Ethiopia* Journal of Nutrition and Metabolism, 2021. 2021. 10.1155/2021/4898970 PMC7817239 33520306

[42] Anwar, S., P. Deshmukh, and B. Garg, *Epidemiological Correlates of Nutritional Anemia in adolescent girls of rural Wardha*. Indian Journal of Community Medicine, 2006. 31.

[43] Kishore, J., *National Health Programs of India. In: Century publications: New Delhi*. 2006. p. p. 82–4.

[44] Motbainor, A., A. Worku, and A. Kumie, *Stunting is associated with food diversity while wasting with food insecurity among underfive children in East and West Gojjam Zones of Amhara Region, Ethiopia*. PLoS ONE, 2015. 10(8). 10.1371/journal.pone.0133542 PMC4540277 26285047

[45] Rose, E.S., et al., *Determinants of undernutrition among children aged 6 to 59 months in rural Zambézia Province, Mozambique: results of two population-based serial cross-sectional surveys*. BMC Nutrition, 2015. 1(1). 10.1186/s40795-015-0039-1 PMC4864006 27182448

[46] Ali D (2013). Household food insecurity is associated with higher child undernutrition in Bangladesh, Ethiopia, and Vietnam, but the effect is not mediated by child dietary diversity. Journal of Nutrition.

[47] Swindale, A., and Paula Bilinsky, *Household Dietary Diversity Score (HDDS) for Measurement of Household Food Access: Indicator Guide (v.2). Washington, D.C.: FHI 360/FANTA* 2006.

[48] Belay E (2019). Prevalence and determinants of pre-adolescent (5–14 years) acute and chronic undernutrition in Lay Armachiho District, Ethiopia. Int J Equity Health.

[49] Damie, T., M. kbebew, and A. Teklehaymanot, *Nutritional status and associated factors among school adolescent in Chiro Town, West Hararge, Ethiopia*. Gaziantep Medical Journal, 2015. 21.

[50] Farella Guzzo, M. and G. Gobbi, *Parental Death During Adolescence: A Review of the Literature* Omega (Westport), 2021: p. 302228211033661. 10.1177/00302228211033661 34324402

[51] Das JK (2017). Nutrition in adolescents: physiology, metabolism, and nutritional needs. Ann N Y Acad Sci.

[52] Manna I (2022). Growth Development and Maturity in Children and Adolescent: relation to Sports and physical activity. American Journal of Sports Science and Medicine.

[53] Nichols MS (2011). Decreasing trends in overweight and obesity among an australian population of preschool children. Int J Obes (Lond).

[54] Rokholm B, Baker JL, Sørensen TI (2010). The levelling off of the obesity epidemic since the year 1999–a review of evidence and perspectives. Obes Rev.

[55] Tariku A (2019). Stunting and its determinants among adolescent girls: findings from the Nutrition Surveillance Project, Northwest Ethiopia. Ecology of Food and Nutrition.

[56] Engidaw MT, Gebremariam AD (2019). Prevalence and associated factors of stunting and thinness among adolescent somalian refugee girls living in eastern somali refugee camps, somali regional state, Southeast Ethiopia. Conflict and Health.

[57] Tariku, E.Z., et al., *Prevalence and factors associated with stunting and thinness among school-age children in Arba Minch Health and demographic surveillance site, Southern Ethiopia*. PLoS ONE, 2018. 13(11). 10.1371/journal.pone.0206659 PMC6214544 30388149

[58] Getachew, T. and A. Argaw, *Intestinal helminth infections and dietary diversity score predict nutritional status of urban schoolchildren from southern Ethiopia*. BMC Nutrition, 2017. 3(1).

[59] Darapheak, C., et al., *Consumption of animal source foods and dietary diversity reduce stunting in children in Cambodia*. International Archives of Medicine, 2013. 6(1). 10.1186/1755-7682-6-29 PMC3720190 23866682

