# Supplementary material for: A systematic review and meta-analysis of the association of dietary diversity with undernutrition in school-aged children
Source: BMC Pediatr. 2023 May 29;23:269. doi: 10.1186/s12887-023-04032-y (PMC10226245; doi:10.1186/s12887-023-04032-y)
Supplement: Supplementary file 1 — Supplementary Material 1 [file 12887_2023_4032_MOESM1_ESM.pdf]

Supplementary Material Content

A systematic review and meta-analysis of the association of dietary diversity with undernutrition in school-aged children.

Table S1. Search strategy and terms.

Table S2. Quality assessment for individual studies included in the meta-analysis.

Table S3. GRADE evidence table for the association dietary diversity and undernutrition.

Table S4: List of excluded studies with the exclusion results.

Figure S1. Funnel plot for the association between dietary diversity and stunting.

Figure S2. Funnel plot for the association between dietary diversity and thinness.

Figure S3. Meta influence for the association between dietary diversity and stunting.

Figure S4. Meta influence for the association between dietary diversity and thinness.

Table S1. Search strategy and terms.

**Database**	**PubMed (n=736)**	**Scopus (n=3969)**	**Web of science (n=771)**
**Date**	8th August 2022	8th August 2022	8th August 2022
**strategy**	((#1) AND (2))	((#1) AND (2))	((#1) AND (2))
**#1**	("Dietary diversity"[All Fields] OR "dietary diversity score"[All Fields] OR "dietary score"[All Fields] OR "DDS"[All Fields] OR "diversity score"[All Fields] OR "diet diversity"[All Fields] OR "household dietary diversity"[All Fields])	(ALL ("Dietary diversity") OR ALL ("dietary diversity score") OR ("DDS") OR ALL ("diversity score") OR ALL ("diet diversity") OR ALL ("household dietary diversity"))	TS=("Dietary diversity" OR "dietary diversity score" OR "DDS" OR "diversity score" OR "diet diversity" OR "household dietary diversity")
**#2**	("wasting"[All Fields] OR ("growth disorders"[MeSH Terms] OR "growth disorders"[All Fields] OR "stunting"[All Fields] OR "stunted"[All Fields]) OR ("malnutrition"[MeSH Terms] OR "malnutrition"[All Fields] OR "malnutrition s"[All Fields] OR "malnutritional"[All Fields] OR "malnutritions"[All Fields]) OR "under nutrition"[All Fields] OR "nutrition disorder"[All Fields] OR ("thinness"[MeSH Terms] OR ("wasting"[All Fields] OR ("growth disorders"[MeSH Terms] OR "growth disorders"[All Fields] OR "stunting"[All Fields] OR "stunted"[All Fields]) OR ("malnutrition"[MeSH Terms] OR "malnutrition"[All Fields] OR "malnutrition s"[All Fields] OR "malnutritional"[All Fields] OR "malnutritions"[All Fields]) OR "under nutrition"[All Fields] OR "nutrition disorder"[All Fields] OR ("thinness"[MeSH Terms] OR "thinness"[All Fields] OR "underweight"[All Fields] OR "underweights"[All Fields]) OR ("growth disorders"[MeSH Terms] OR ("growth"[All Fields] AND "disorders"[All Fields]) OR "growth disorders"[All Fields] OR "stunting"[All Fields] OR "stunted"[All Fields]) OR "stunted growth"[All Fields] OR ("body height"[MeSH Terms] OR ("body"[All Fields]	(ALL ("wasting") OR ALL ("growth disorders") OR ALL ("stunting") OR ALL ("stunted") OR ALL ("growth disorder") OR ALL ("malnutrition") OR ALL ("under nutrition") OR ALL ("nutrition disorder") OR ALL ("thinness") OR ALL ("underweight") OR ALL ("stunted growth") OR ALL ("body height") OR ALL ("height for age") OR ALL ("weight for height") OR ALL ("weight for age"))	TS=("wasting" OR "growth disorders" OR "stunting" OR "stunted" OR "growth disorder" OR "malnutrition" OR "under nutrition" OR "nutrition disorder" OR "thinness" OR "underweight" OR "stunted growth" OR "body height" OR "height for age" OR "weight for height" OR "weight for age")

Table S2. Quality assessment for individual studies included in the meta-analysis.

Reference	Selection				Comparability	Outcome		score
Author, year	Representativeness of the sample	Sample size	Non-respondents	Ascertainment of the exposure	Confounding factors are controlled	Assessment of the outcome	Statistical test	Total
**Adeomi et al. 2022**	1	1	1	2	2	2	1	10
**Mulu Birru et al 2021**	1	1	1	2	2	2	1	10
**Mersha et al 2021**	1	1	1	2	2	2	1	10
**Patill et al. 2021**	1	1	0	2	0	2	1	7
**Yasuoka et al 2020**	1	1	1	2	2	0	0	8
**Shiferaw et al 2020**	1	1	1	2	0	1	1	7
**Kahssay et al 2020**	1	1	1	2	2	2	1	10
**Gezahegn et al 2020**	1	1	1	2	2	2	1	10
**Tariku et al 2020**	1	1	1	2	0	2	0	7
**Jikamo et al 2019**	1	1	1	2	2	2	1	10
**Getaneh et al 2019**	1	1	1	2	2	2	1	10
**Engidaw et al 2019**	1	1	0	2	1	2	1	8
**Belay et al 2019**	1	1	1	2	2	0	1	8
**Aiga et al 2019**	1	1	1	2	2	2	1	10
**Tariku et al 2018**	1	1	1	2	2	2	1	10
**Radhika et al 2018**	1	1	1	2	1	0	1	8
**Getachew et al 2017**	1	1	1	2	2	2	1	10
**Wassie et al 2015**	1	1	1	2	2	2	1	10
**Darapheak et al 2013**	1	0	1	2	2	2	1	9
**Niranjala et al 2011**	1	0	1	2	2	2	1	9

Table S3. GRADE evidence table for the association dietary diversity and undernutrition.

Certainty assessment							No of patients	Effect		Certainty	Importance
**No of studies**	**Study design**	**Risk of bias**	**Inconsistency**	**Indirectness**	**Imprecision**	**Other considerations**	**population**	**Relative (95% CI)**	**Absolute (95% CI)**		
**Stunting**											
13	observational studies	not serious	not serious	not serious	Serious [a]	publication bias strongly suspected	8539	**OR 1.43** (1.08 to 1.89)	**1 fewer per 1,000** (from 2 fewer to 1 fewer)	⊕◯◯◯ Very low	IMPORTANT
**Thinness**											
10	observational studies	not serious	Serious [b]	not serious	very serious [a]	none	9434	**OR 1.10** (0.81 to 1.49)	**1 fewer per 1,000** (from 1 fewer to 1 fewer)	⊕◯◯◯ Very low	IMPORTANT
**Wasting**											
2	observational studies	not serious	not serious	not serious	not serious	strong association	1004	**OR 2.18** (1.41 to 3.36)	**2 fewer per 1,000** (from 3 fewer to 1 fewer)	⊕⊕⊕◯ Moderate	IMPORTANT

CI: confidence interval; **OR:** odds ratio

Explanation:

a : Serious imprecision since the 95% confidence intervals includes no effect (RR of 1.00), but fails to exclude important harm (RR of >1.25). Downgraded.

b: Serious inconsistency since I^2 = 79%, Phet <0.001, that was largely unexplained in pre-specified subgroup and sensitivity analyses. Downgraded.

Table S4: List of excluded studies with the exclusion results.

list of studies excluded	exclusion reason.
Geda et al, 2021[1], Yoseph et al, 2020 [2] Workie et al, 2020 [3], Trisasmita et al, 2020 [4], Sheikh et al, 2020 [5], Rakotomananaet al, 2020 [6], Mutuku et al, 2020 [7], Modjadji et al, 2020 [8], Li et al, 2020 [9], Guyatt, et al, 2020 [10], Boulom et al, 2020 [11], Bogale, et al, 2020 [12], Walters et al, 2019 [13], Wondimagegne et al, 2019 [14], Nai et al, 2019 [15], Mya et al, 2019 [16], Mohammed et al, 2019 [17], Meshram et al, 2019 [18], Malako et al, 2019 [19], Kim et al, 2019 [20], Khamis et al, 2019 [21], Kang et al, 2019 [22], Hein et al, 2019 [23], Girma et al, 2019 [24], Bosha et al, 2019 [25], Sié et al, 2018 [4126], MakamtoSobgui et al, 2018 [27], Harding et al, 2018 [28], Gosdin et al, 2018 [29], Campbell et al, 2018 [30], Borkotoky et al, 2018 [31], Berhanu et al, 2018 [32], Ahmad et al, 2018 [33], Aditi Krishna et al, 2018 [34], Wondafrash et al, 2017 [35], Wang et al, 2017 [36], Tariku et al, 2017 [37], Tariku et al, 2017 [38], Mahmudiono et al, 2017 [39], Krasevec et al, 2017 [40], Kim et al, 2017 [41], Choudhury et al, 2017 [42], Chandrasekhar et al, 2017 [43], Balalian et al, 2017 [44], Ali et al, 2017 [45], Abate et al, 2017 [46], Udoh et al, 2016 [47], Tariku et al, 2016 [48], Ocampo-Guirindola et al, 2016 [49], Maradzika et al, 2016 [50], Altare et al, 2016 [51], Aguayo et al, 2016 [52], Rose et al, 2015 [53], McDonald et al, 2015 [54], Fekadu et al, 2015 [55], Bentley et al, 2015 [56], Bukania et al, 2014 [57], Rah et al, 2010 [58], Marriott et al, 2010 [59], Nurhayati et al, 2020 [60], Hintsa et al, 2019 [61], Berhe et al, 2019 [62], Paudel et al, 2012 [63], Belayneh et al, 2020 [64], Islam et al, 2018 , Kamenju et al, 2017 [65]	All these studies were done in the range of under 5 years.

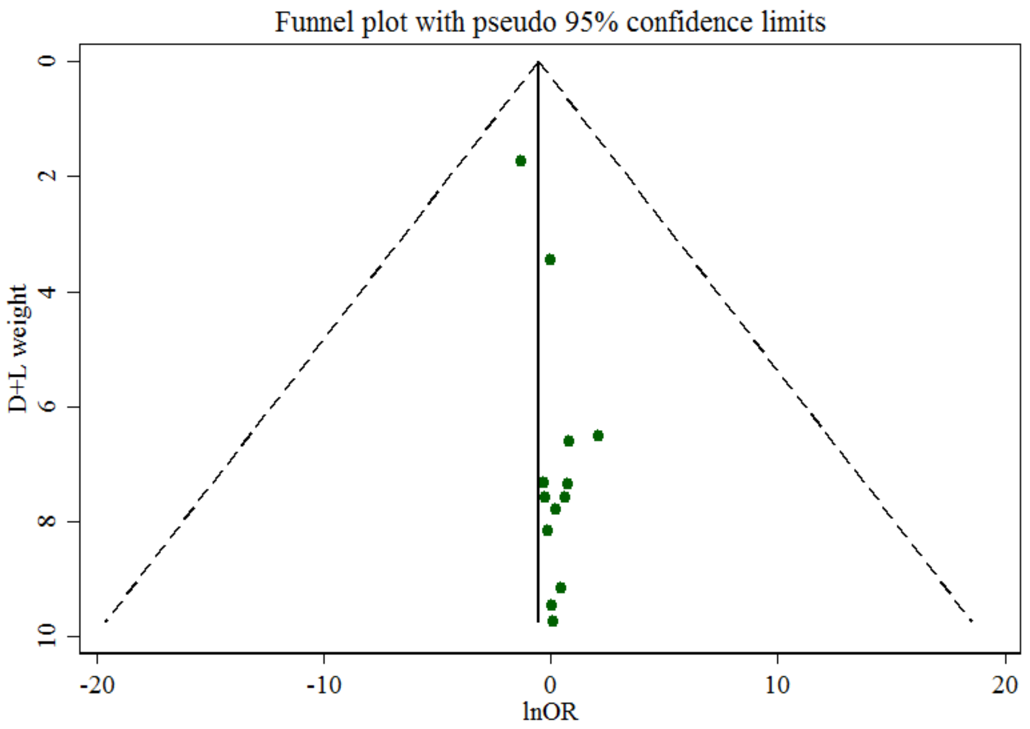

Figure S1. Funnel plot for the association between dietary diversity and stunting.

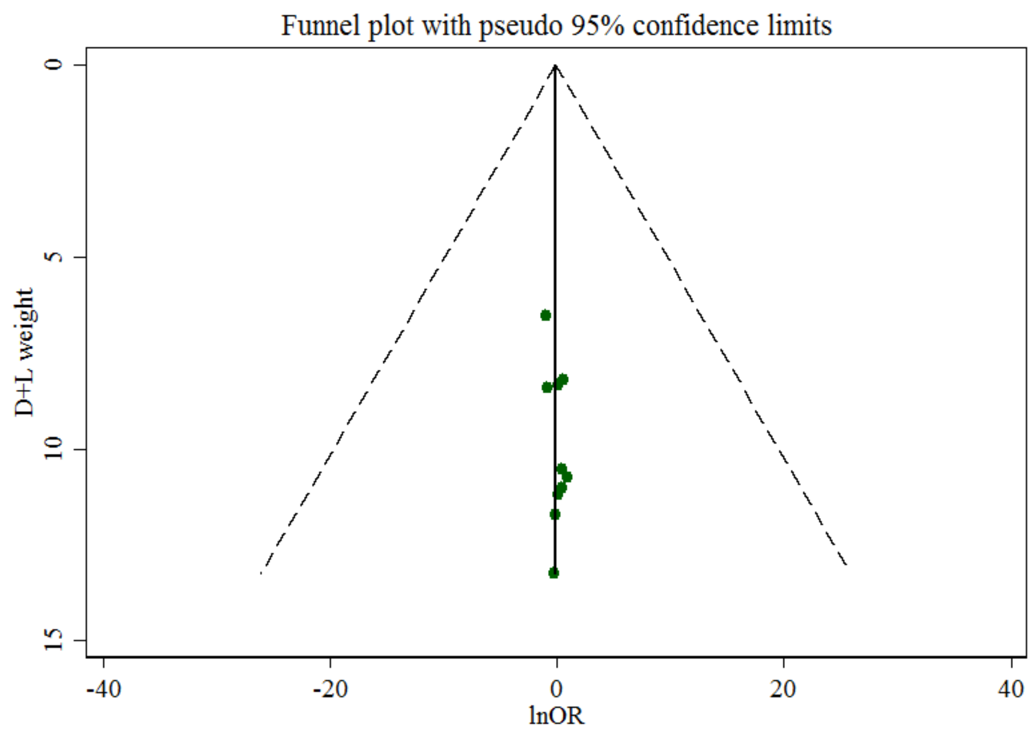

Figure S2. Funnel plot for the association between dietary diversity and thinness.

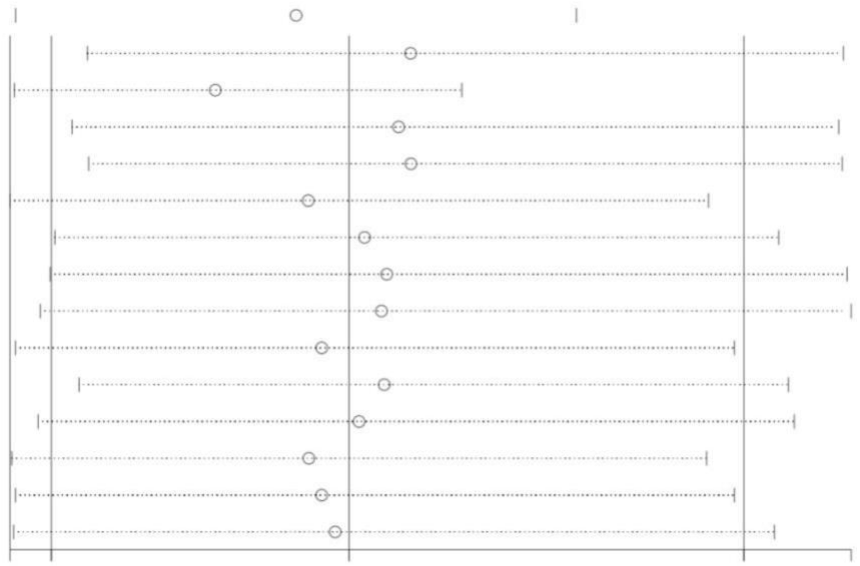

Figure S3. Meta influence for the association between dietary diversity and stunting.

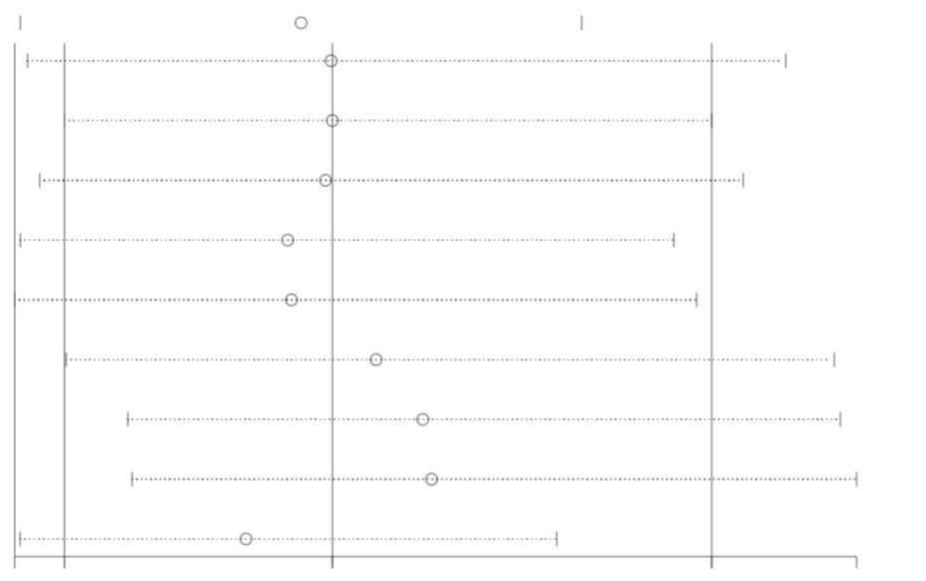

Figure S4. Meta influence for the association between dietary diversity and thinness.

1. Geda, N.R., et al., *Multiple anthropometric and nutritional deficiencies in young children in Ethiopia: a multi-level analysis based on a nationally representative data.* BMC Pediatrics, 2021. **21**(1).
2. Yoseph, A. and H. Beyene, *The high prevalence of intestinal parasitic infections is associated with stunting among children aged 6-59 months in Boricha Woreda, Southern Ethiopia: A cross-sectional study.* BMC Public Health, 2020. **20**(1).
3. Workie, S.B., et al., *Level of Undernutrition and Its Determinants Among Children Aged 12-59 Months in Wolaita District, Ethiopia.* Pediatric Health Med Ther, 2020. **11**: p. 109-117.
4. Trisasmita, L., et al., *Identification of dietary diversity associated with stunting in Indonesia.* Malaysian Journal of Nutrition, 2020. **26**(1): p. 85-92.
5. Sheikh, N., et al., *Infant and young child feeding practice, dietary diversity, associated predictors, and child health outcomes in Bangladesh.* Journal of Child Health Care, 2020. **24**(2): p. 260-273.
6. Rakotomanana, H., et al., *Maternal Knowledge, Attitudes, and Practices of Complementary Feeding and Child Undernutrition in the Vakinankaratra Region of Madagascar: A Mixed-Methods Study.* Current Developments in Nutrition, 2020. **4**(11).
7. Mutuku, J.N., S. Ochola, and J. Osero, *Maternal knowledge and complementary feeding practices and their relationship with nutritional status among children 6-23 months old in pastoral community of marsabit county, kenya: A cross-sectional study.* Current Research in Nutrition and Food Science, 2020. **8**(3): p. 862-876.
8. Modjadji, P., D. Molokwane, and P.O. Ukegbu, *Dietary Diversity and Nutritional Status of Preschool Children in North West Province, South Africa: A Cross Sectional Study.* Children (Basel), 2020. **7**(10).
9. Li, Z., et al., *Factors Associated With Child Stunting, Wasting, and Underweight in 35 Low- and Middle-Income Countries.* JAMA network open, 2020. **3**(4): p. e203386.
10. Guyatt, H., et al., *Prevalence and predictors of underweight and stunting among children under 2 years of age in Eastern Kenya.* Public Health Nutrition, 2020. **23**(9): p. 1599-1608.
11. Boulom, S., et al., *Factors associated with child malnutrition in mountainous ethnic minority communities in Lao PDR.* Global Health Action, 2020. **13**(sup2).
12. Bogale, B., B.T. Gutema, and Y. Chisha, *Prevalence of Stunting and Its Associated Factors among Children of 6-59 Months in Arba Minch Health and Demographic Surveillance Site (HDSS), Southern Ethiopia: A Community-Based Cross-Sectional Study.* Journal of Environmental and Public Health, 2020. **2020**.
13. Walters, C.N., et al., *Maternal determinants of optimal breastfeeding and complementary feeding and their association with child undernutrition in Malawi (2015-2016).* BMC Public Health, 2019. **19**(1).
14. Wondimagegne, Z., et al., *Child Feeding Practice and Primary Health Care as Major Correlates of Stunting and Underweight among 6- to 23-Month-Old Infants and Young Children in Food-Insecure Households in Ethiopia.* Current developments in nutrition, 2020. **4**: p. nzaa137.
15. Nai, H.M.E. and B.S. Renyoet, *Dietary Diversity Is Associated with Stunting among Children 6-23 months in Area of Mergangsan Public Health Center, Yogyakarta.* Annals of Nutrition and Metabolism, 2019. **75**: p. 333-333.
16. Mya, K.S., A.T. Kyaw, and T. Tun, *Feeding practices and nutritional status of children age 6-23 months in Myanmar: A secondary analysis of the 2015-16 Demographic and Health Survey.* PLoS ONE, 2019. **14**(1).
17. Mohammed, S.H., B. Larijani, and A. Esmaillzadeh, *Concurrent anemia and stunting in young children: Prevalence, dietary and non-dietary associated factors.* Nutrition Journal, 2019. **18**(1).
18. Meshram, I.I., et al., *Infant and young child feeding practices, sociodemographic factors and their association with nutritional status of children aged <3 years in India: Findings of the National Nutrition Monitoring Bureau survey, 2011-2012.* Public Health Nutrition, 2019. **22**(1): p. 104-114.
19. Malako, B.G., et al., *Stunting and anemia among children 6-23 months old in Damot Sore district, Southern Ethiopia.* BMC Nutrition, 2019. **5**(1).
20. Kim, R., et al., *Assessing associational strength of 23 correlates of child anthropometric failure: An econometric analysis of the 2015-2016 National Family Health Survey, India.* Social Science and Medicine, 2019. **238**.
21. Khamis, A.G., et al., *The influence of dietary diversity on the nutritional status of children between 6 and 23 months of age in Tanzania.* BMC Pediatrics, 2019. **19**(1).
22. Kang, Y. and J. Kim, *Risk factors for undernutrition among children 0–59 months of age in Myanmar.* Maternal and Child Nutrition, 2019. **15**(4).
23. Hein, A.K., et al., *Dietary Diversity, Social Support and Stunting among Children Aged 6-59 Months in an Internally Displaced Persons Camp in Kayin State, Myanmar.* Clin Nutr Res, 2019. **8**(4): p. 307-317.
24. Girma, A., et al., *Undernutrition and associated factors among urban children aged 24-59 months in Northwest Ethiopia: A community based cross sectional study.* BMC Pediatrics, 2019. **19**(1).
25. Bosha, T., et al., *Dietary Diversity and Anthropometric Status of Mother-Child Pairs from Enset (False Banana) Staple Areas: A Panel Evidence from Southern Ethiopia.* Int J Environ Res Public Health, 2019. **16**(12).
26. Sié, A., et al., *Dietary diversity and nutritional status among children in rural Burkina Faso.* International Health, 2018. **10**(3): p. 157-162.
27. Makamto Sobgui, C., et al., *Predictors of poor nutritional status among children aged 6-24 months in agricultural regions of Mali: A cross-sectional study.* BMC Nutrition, 2018. **4**(1).
28. Harding, K.L., V.M. Aguayo, and P. Webb, *Birthweight and feeding practices are associated with child growth outcomes in South Asia.* Maternal and Child Nutrition, 2018. **14**.
29. Gosdin, L., et al., *The co-occurrence of anaemia and stunting in young children.* Maternal and Child Nutrition, 2018. **14**(3).
30. Campbell, R.K., et al., *Infant and young child feeding practices and nutritional status in Bhutan.* Maternal and Child Nutrition, 2018. **14**.
31. Borkotoky, K., S. Unisa, and A.K. Gupta, *State-level dietary diversity as a contextual determinant of nutritional status of children in India: A multilevel approach.* Journal of Biosocial Science, 2018. **50**(1): p. 26-52.
32. Berhanu, G., S. Mekonnen, and M. Sisay, *Prevalence of stunting and associated factors among preschool children: A community based comparative cross sectional study in Ethiopia.* BMC Nutrition, 2018. **4**(1).
33. Ahmad, I., et al., *Dietary diversity and stunting among infants and young children: A cross-sectional study in Aligarh.* Indian Journal of Community Medicine, 2018. **43**(1): p. 34-36.
34. Aditi Krishna 1 , I.M.-G., Mark McGovern 4 5 , Víctor M Aguayo 6 , S V Subramanian 7 8, *Trends in inequalities in child stunting in South Asia.* Matern Child Nutr, 2018.
35. Wondafrash, M., et al., *Feeding practices and growth among young children during two seasons in rural Ethiopia.* BMC Nutrition, 2017. **3**(1).
36. Wang, A., et al., *The dietary diversity and stunting prevalence in minority children under 3 years old: A cross-sectional study in forty-two counties of Western China.* British Journal of Nutrition, 2017. **118**(10): p. 840-848.
37. Tariku, A., et al., *Stunting and its determinant factors among children aged 6-59 months in Ethiopia.* Italian Journal of Pediatrics, 2017. **43**(1).
38. Tariku, A., et al., *Child wasting is a severe public health problem in the predominantly rural population of Ethiopia: A community based cross-sectional study.* Archives of Public Health, 2017. **75**(1).
39. Kawade, R., *Zinc status and its association with the health of adolescents: A review of studies in India.* Global Health Action, 2012. **5**(1).
40. Krasevec, J., et al., *Diet quality and risk of stunting among infants and young children in low- and middle-income countries.* Maternal and Child Nutrition, 2017. **13**.
41. Kim, R., et al., *Relative importance of 13 correlates of child stunting in South Asia: Insights from nationally representative data from Afghanistan, Bangladesh, India, Nepal, and Pakistan.* Social Science and Medicine, 2017. **187**: p. 144-154.
42. Choudhury, N., et al., *Determinants of age-specific undernutrition in children aged less than 2 years—the Bangladesh context.* Maternal and Child Nutrition, 2017. **13**(3).

43. Chandrasekhar, S., et al., *Household food insecurity and children's dietary diversity and nutrition in India. Evidence from the comprehensive nutrition survey in Maharashtra.* Maternal and Child Nutrition, 2017. **13**.
44. Balalian, A.A., et al., *Prevalence and determinants of stunting in a conflict-ridden border region in Armenia - A cross-sectional study.* BMC Nutrition, 2017. **3**(1).
45. Ali, Z., et al., *The effect of maternal and child factors on stunting, wasting and underweight among preschool children in Northern Ghana.* BMC Nutrition, 2017. **3**(1).
46. Abate, K.H. and T. Belachew, *Care and not wealth is a predictor of wasting and stunting of 'The Coffee Kids' of Jimma Zone, Southwest Ethiopia.* Nutrition and Health, 2017. **23**(3): p. 193-202.
47. Udoh, E.E. and O.K. Amodu, *Complementary feeding practices among mothers and nutritional status of infants in Akpabuyo Area, Cross River State Nigeria.* SpringerPlus, 2016. **5**(1).
48. Tariku, A., et al., *Nearly half of preschool children are stunted in Dembia district, Northwest Ethiopia: A community based cross-sectional study.* Archives of Public Health, 2016. **74**(1).
49. Ocampo-Guirindola, M.L., et al., *Association between dietary diversity score and nutritional status of Filipino children aged 6-23 months.* Philippine Journal of Science, 2016. **145**(1): p. 57-69.
50. Adeyemo, A.A., et al., *ZRANB3 is an African-specific type 2 diabetes locus associated with beta-cell mass and insulin response.* Nature Communications, 2019. **10**(1).
51. Altare, C., et al., *Factors associated with stunting among pre-school children in southern highlands of Tanzania.* Journal of Tropical Pediatrics, 2016. **62**(5): p. 390-408.
52. Aguayo, V.M., et al., *Determinants of stunting and poor linear growth in children under 2 years of age in India: An in-depth analysis of Maharashtra's comprehensive nutrition survey.* Maternal and Child Nutrition, 2016. **12**: p. 121-140.
53. Rose, E.S., et al., *Determinants of undernutrition among children aged 6 to 59 months in rural Zambézia Province, Mozambique: Results of two population-based serial cross-sectional surveys.* BMC Nutrition, 2015. **1**(1).
54. McDonald, C.M., et al., *Household food insecurity and dietary diversity as correlates of maternal and child undernutrition in rural Cambodia.* European Journal of Clinical Nutrition, 2015. **69**(2): p. 242-246.
55. Fekadu, Y., et al., *Factors associated with nutritional status of infants and young children in Somali Region, Ethiopia: A cross- sectional study Global health.* BMC Public Health, 2015. **15**(1).
56. Bentley, A., et al., *Malnutrition and infant and young child feeding in informal settlements in mumbai, india: Findings from a census.* Food Science and Nutrition, 2015. **3**(3): p. 257-271.
57. Bukania, Z.N., et al., *Food insecurity and not dietary diversity is a predictor of nutrition status in children within semiarid agro-ecological zones in Eastern Kenya.* Journal of Nutrition and Metabolism, 2014. **2014**.
58. Rah, J.H., et al., *Low dietary diversity is a predictor of child stunting in rural Bangladesh.* European Journal of Clinical Nutrition, 2010. **64**(12): p. 1393-1398.
59. Marriott, B.P., et al., *How well are infant and young child World Health Organization (WHO) feeding indicators associated with growth outcomes? An example from Cambodia.* Maternal and Child Nutrition, 2010. **6**(4): p. 358-373.
60. Nurhayati, E., et al., *Dietary diversity, vitamin D intake and childhood stunting: A case-control study in Bantul, Indonesia.* Malaysian Journal of Nutrition, 2020. **26**(2): p. 273-287.
61. Hintsa, S. and K. Gereziher, *Determinants of underweight among 6-59 months old children in Berahle, Afar, North East Ethiopia: a case control study 2016.* BMC Research Notes, 2019. **12**(1).
62. Berhe, K., et al., *Risk factors of stunting (chronic undernutrition) of children aged 6 to 24 months in Mekelle City, Tigray Region, North Ethiopia: An unmatched case-control study.* PLoS ONE, 2019. **14**(6).
63. Paudel, R., et al., *Risk factors for stunting among children: A community based case control study in Nepal.* Kathmandu University Medical Journal, 2012. **10**(39): p. 18-24.
64. Belayneh, M., E. Loha, and B. Lindtjørn, *Seasonal Variation of Household Food Insecurity and Household Dietary Diversity on Wasting and Stunting among Young Children in A Drought Prone Area in South Ethiopia: A Cohort Study.* Ecology of Food and Nutrition, 2020: p. 1-26.
65. Kamenju, P., et al., *Nutritional status and complementary feeding among HIV-exposed infants: a prospective cohort study.* Maternal and Child Nutrition, 2017. **13**(3).
